# Supplementary material for: Word and bit line operation of a 1 × 1 μm2 superconducting vortex-based memory
Source: Nat Commun. 2023 Aug 15;14:4926. doi: 10.1038/s41467-023-40654-7 (PMC10427686; doi:10.1038/s41467-023-40654-7)
Supplement: Supplementary file 1 — Supplementary Information [file 41467_2023_40654_MOESM1_ESM.pdf]

Supplementary information to the manuscript:
Word and bit line operation of a $1 \times 1\ \mu m^2$ superconducting vortex-based memory

I. Characteristics of 5 μm cells

Supplementary Figure 1 shows the current-voltage characteristics of cells with $L_x \sim 5\ \mu$m. Fig. 1 (a) shows the temperature variation of I-Vs at zero field. In Fig. 1 (b) the I-Vs at four magnetic fields and a fixed T are shown. Typical resistively-shunted Josephson junctions behavior is observed. All studied JJs show similar characteristics.

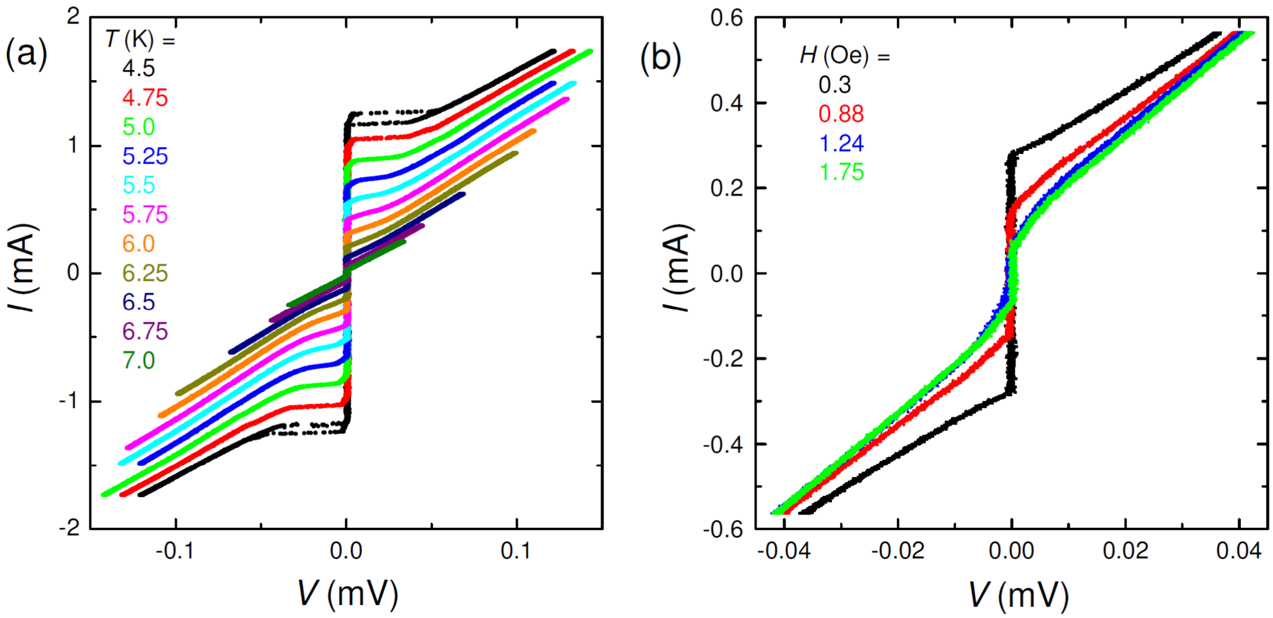

Supplementary Figure 1.
The current-voltage characteristics of Josephson junctions on two AVRAM cells with $L_x \sim 5\ \mu$m. (a) A set of I-V characteristics at different temperatures and $H \simeq 0$. (b) A set of I-Vs at four magnetic fields and $T = 6.65$ K (for another junction with a nominal depth of the groove 50 nm).

Supplementary Figure 2 (a) shows a SEM image of the largest cell with $L_x \simeq 5$ μm on the same chip, as presented in the manuscript. Figs. 2 (b-c) show measured $I_c(H)$ modulations for both JJs on this cell (b) without a vortex, (c) with a trapped vortex and (d) with a trapped antivortex.

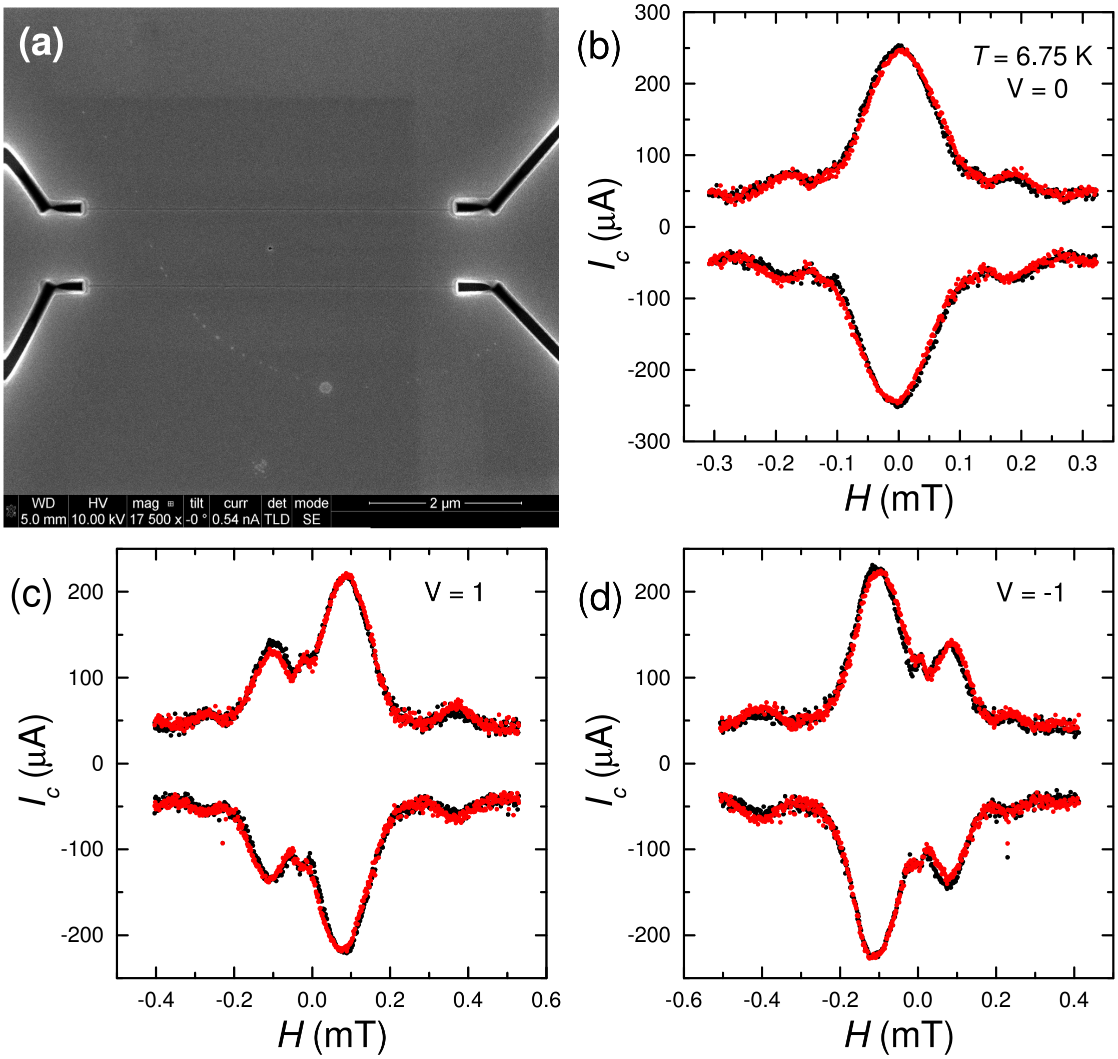

Supplementary Figure 2. **Characteristics of a larger cell on the same chip.** (a) SEM image of the cell with $L_x = 5$ μm on the same chip, as studied in the manuscript. (b-d) Measured $I_c(H)$ modulations for both JJs on this cell (black and red symbols): (b) without a trapped vortex, V=0; (c) with a trapped vortex, V=1; and (d) with an antivortex, V=-1. All measurements were performed at $T = 6.75$ K.

II. Flux quantization field in planar junctions

From the comparison of $I_c(H)$ patterns in Figs. 1 (c), (d) and Fig. S2 (b) it can be seen that the flux quantization field, ΔH, is increasing with decreasing junction length. $\Delta H = \Phi_0/A$, is determined by the effective flux quantization area, A. For our planar junctions with dissimilar electrodes (the outer electrode is very long and the inner is narrow) it is equal to $A = \left[L_x^2/1.8 + L_x L_z/2\right]/2$ [33]. Here L_z is the effective width of the inner electrode. Since this electrode is shared by both JJs, $2L_z = w$, where w is the separation between JJs, $w \simeq 1$ μm. Thus,

$$\Delta H = 2\Phi_0 \left[\frac{L_x^2}{1.8} + \frac{L_x w_z}{4}\right]^{-1}. \qquad (S1)$$

This expression describes very well the observed flux-quantization fields.

III. Test junction response for the 1×1 μm^2 cell.

The 1×1 μm^2 cell, analyzed in the manuscript contains two JJs. In the manuscript we showed only characteristics of the readout JJ near the vortex trap. The Supplementary Figure 3 shows simultaneously measured responses of both JJs on this cell for the case, corresponding to Fig. 3 (b). It is seen that the resistances of both JJs are correlated, but the response of the test junction is much smaller because of a significantly larger distance to the vortex trap.

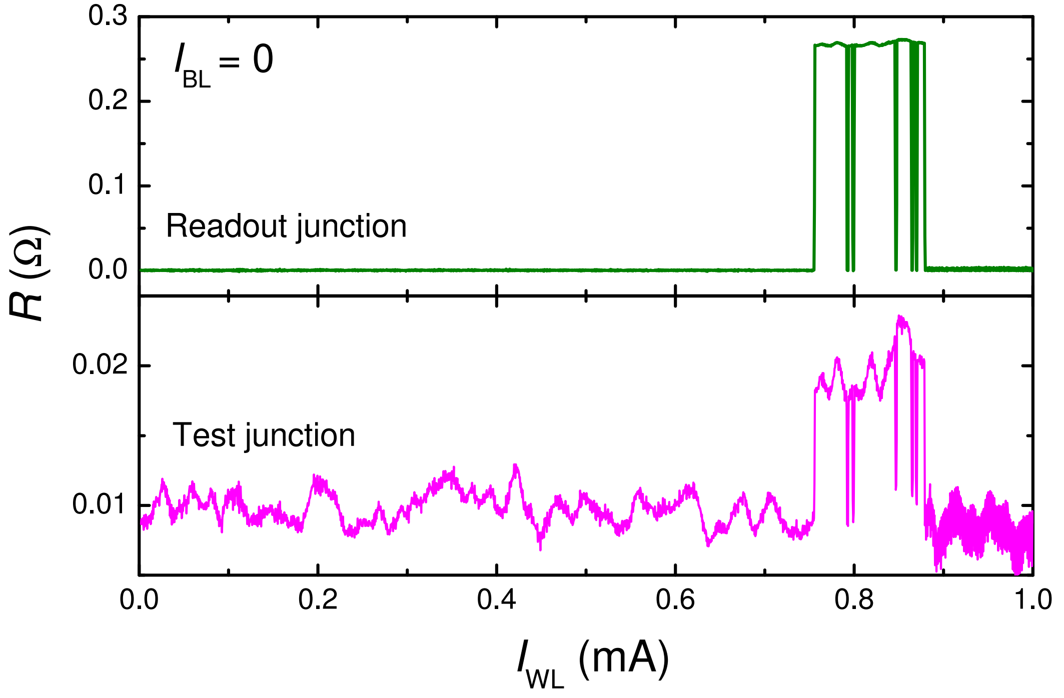

Supplementary Figure 3. **Demonstration of the test junction response.** Simultaneously measured resistances of the readout JJ (top) and the test JJ (bottom) as a function of the wordline pulse amplitude for the case from Fig. 3 (b). A correlated response of both junctions is seen, confirming that the vortex is indeed placed in the trap.

IV. Word and bit-line operation

Supplementary Figure 4 demonstrates measured shapes of current pulses sent via wordline (a) and bitline (b) for the 1×1 μm^2 cell. A single short pulse (in this case with the amplitude +29 μA) is sent through the WL. The bit line current consists of the sinusoidal ac-current at a frequency f = 23 Hz and a short pulse (in this case with the amplitude -230 μA), synchronized with the WL pulse. The BL current passes through the readout JJ, see Fig. 2 (a) and (b). The ac current is used for a continuous readout of the cell state via lock-in measurements of the junction resistance. The amplitude of the ac-current, $I_{ac} = 130$ μA, is such that it is larger than $I_c(\mathrm{V} = \pm 1) \simeq 100$ μA, but smaller than $I_c(\mathrm{V} = 0) \simeq 150$ μA, see Fig. 3 (a). Therefore, the V=0 state has zero resistance and V=±1 states have finite resistances, $R \sim 0.3$ Ω, as shown in Fig. S3. Rightmost panels in Fig. S4 represent closeups on the pulse sequences. It can be seen that the BL pulse is applied at zero of the ac-current. The switching occurs very rapidly at the time scale much shorter than the period of the ac-current ∼ 43 ms. Therefore, the ac current does not interfere with the pulses and does not affect the switching. The programmed pulse widths are ∼ 100 μs. The rightmost panels represent actual measurements of the flowing currents. They are broadened by the time delays in the twisted pairs through which the current pulses are delivered to the sample and the response voltage is transmitted back to the measurement equipment.

Supplementary Figure 5 illustrates the write operation at $I_{\mathrm{BL}} = \pm 0.37$ mA versus I_{WL} of both signs. It can be seen that the switching process is centrosymmetric: $I_{\mathrm{WL}}(I_{\mathrm{BL}}) = -I_{\mathrm{WL}}(-I_{\mathrm{BL}})$. However, the vorticity has opposite signs in the centrosymmetric states, as indicated in the Figure. Such behavior is expected if vortices are following the same route under the influence of Lorentz forces from both currents.

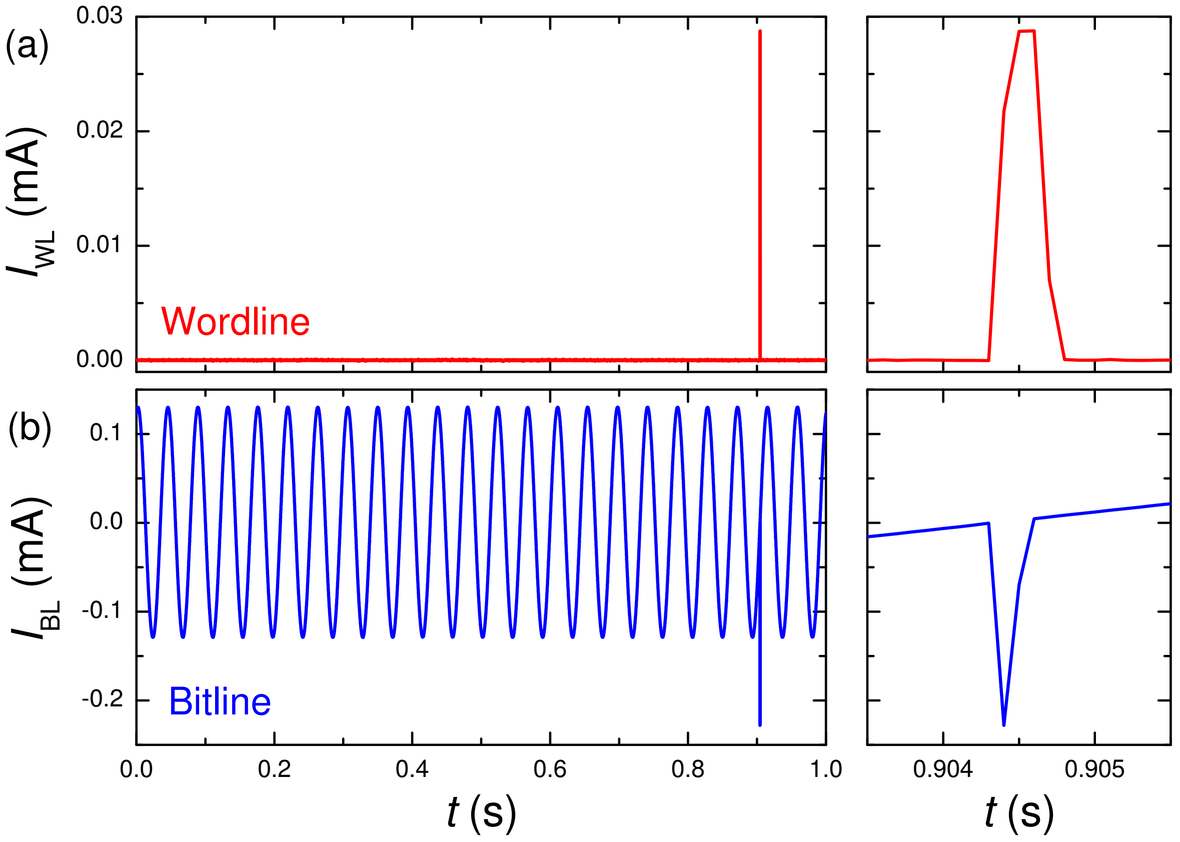

Supplementary Figure 4. **The shapes of pulses sent via word and bit lines in the** $1 \times 1\ \mu\mathrm{m}^2$ **cell.** Panels (a) and (b) show measured time-dependencies of currents flowing through the wordline (a) and bitline (b). The WL contains a single pulse. The BL current is a combination of a sinusoidal current at $f = 23$ Hz, which is used for the continuous readout of the junction resistance and a sharp pulse, synchronized with the WL pulse. Rightmost panels show enlarged views of the two pulses.

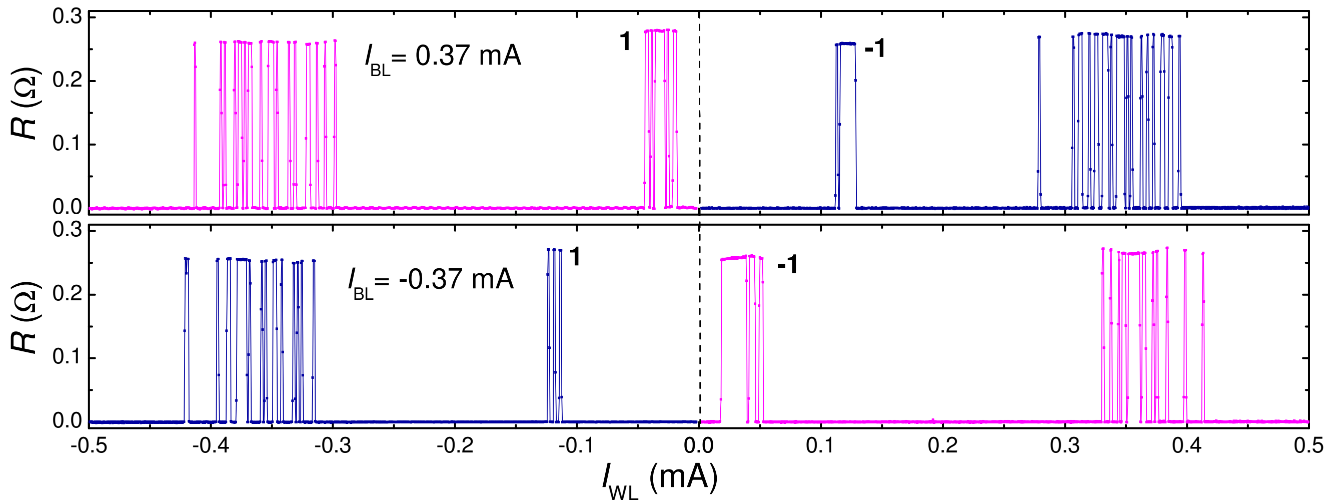

Supplementary Figure 5. **Word and bit-line operation of the** $1 \times 1\ \mu\mathrm{m}^2$ **cell.** The readout junction resistance is shown as a function of the WL pulse amplitude, I_{WL}, for fixed amplitudes of the BL current. $I_{\mathrm{BL}} = \pm 370\ \mu$A and I_{WL} of either directions. The color emphasizes the centrosymmetric response, $R(I_{\mathrm{WL}}, I_{\mathrm{BL}}) = R(-I_{\mathrm{WL}}, -I_{\mathrm{BL}})$, accompanied by the flipping of the vortex sign ($\pm 1 \rightarrow \mp 1$).

V. The origin of cooperative word and bit-line effect.

As discussed in the manuscript, we observed a cooperative WL+BL effect, which led to a significant reduction in the total threshold current. Importantly, this effect was also accompanied by flipping of the sign of trapped vortex. For the bare WL operation, a positive I_{WL} writes a vortex, but for the WL+BL operation, it writes an antivortex, as marked in Supplementary Fig. 5. This indicates that the BL current activates a new easy channel for vortex entrance, not along the track from the bottom of the cell but from the opposite direction, i.e., from the top readout junction (see Fig. 2(a)). Since the flux penetrates via the junction, it most likely involves the creation of a Josephson vortex, which is first moved to the center of the JJ by I_{BL} and then is pushed into the trap by I_{WL}. The positive I_{WL} creates a negative H_y field component at the JJ. The negative I_{BL} creates a positive/negative H_y at the left/right edges of the JJ. Therefore, negative I_{BL} + positive I_{WL} generates a larger negative H_y at the right edge of the JJ. Thus, a Josephson antivortex enters the JJ from the right edge and is pushed to the center of the JJ (to the left) by the negative I_{BL}. The positive I_{WL} pushes the Josephson antivortex downwards and, therefore, leads to $0 \rightarrow -1$ switching.

From the Supplementary Fig. 5 is is seen that the threshold I_{WL} currents for $0 \rightarrow -1$ operation depends on the polarity of I_{BL} and is larger for positive I_{BL}. This indicate the presence of an additional left-right asymmetry in the readout JJ, due to which it is easier for Josephson vortices to enter from the right side of the JJ. Some left/right asymmetry can indeed be seen in the SEM image, Fig. 2(a). This again indicates the importance of conscious geometrical design of such mesoscopic fluxonic quantum dots.

VI. High-endurance operation

Supplementary Figure 5 demonstrates high-endurance operation of the 1×1 μm^2 cell for WL pulses from Figs. 4 (b) and (c).

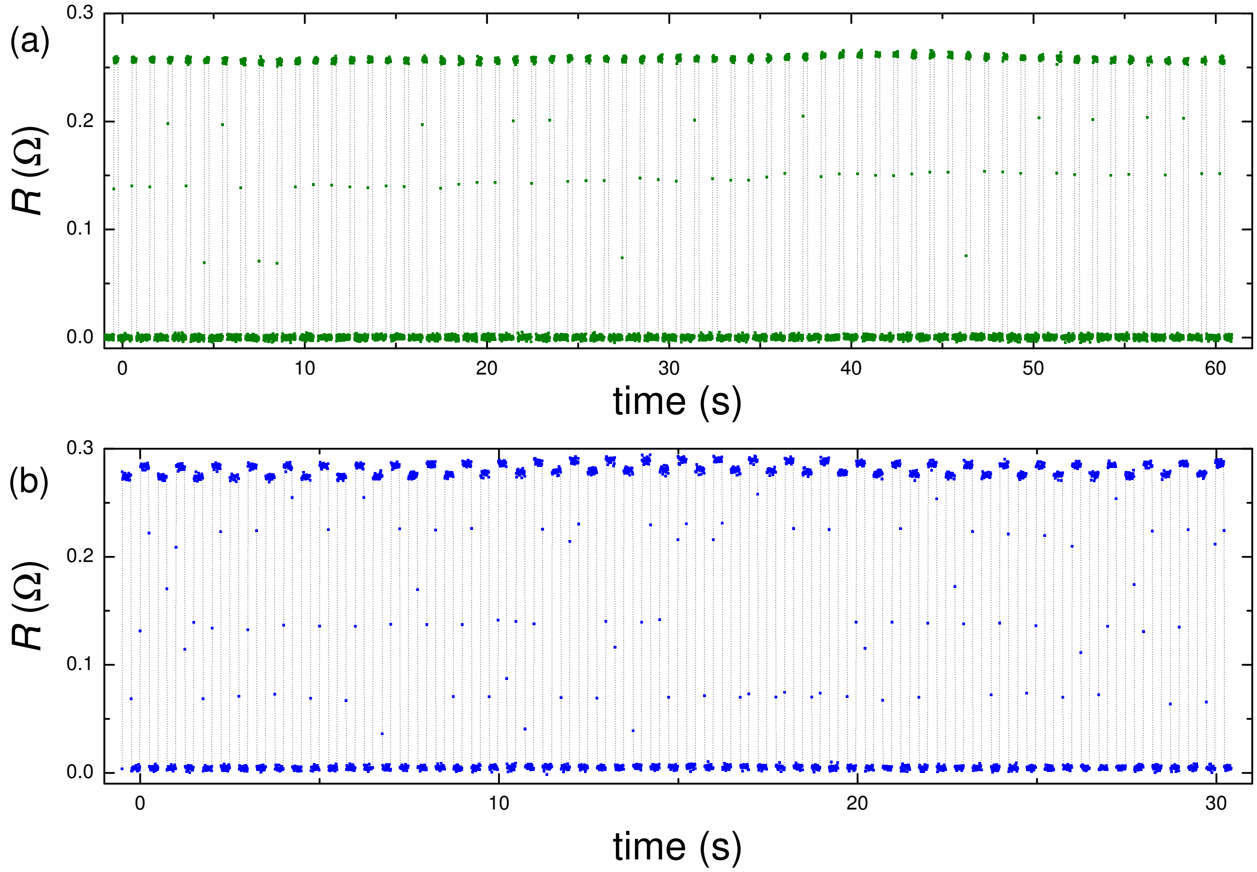

Supplementary Figure 6. **Demonstration of high-endurance operation of the 1×1 μm^2 cell.** Extended time dependence of the readout junction resistance for the following operations: (a) $0 \rightarrow -1$ switching for a WL pulse train from Fig. 4 (b); and (b) $0 \rightarrow -1 \rightarrow 0 \rightarrow 1$ switching for a WL pulse train from Fig. 4 (c).

VII. Stratification of switching diagrams

The presented data shows that switching current diagrams sometimes exhibit stratification, which could reduce the tolerance margins of the device. Fortunately, the stratification can be removed by modest variation of the experimental conditions, as demonstrated in Supplementary Figure 7. Panel (a) shows WL switching diagrams for the $1x1$ μm^2 cell studied in the manuscript, measured at constant $T = 5.85$ K, but at different pulse duration. Panel (b) shows switching diagrams for $L_x = 5$ μm cell, similar to that in Fig. S2 (a), measured with the same pulse duration, but at different temperatures. It can be seen that in both cases the stratification is removed by a modest modifications of experimental conditions.

The likely origin of stratification is related to the long duration of our current pulses (~ 100 μs) compared to the vortex time of flight in the cell (sub-ns). Due to this, at high current amplitudes, the device goes into the flux-flow state with many vortices passing through the device. This can lead to the formation of phase-slip line – a hot track along which vortices and antivortices periodically enter from opposite sides of the device and kick out or annihilate an already trapped vortex. The latter is clear from the $1 \rightarrow -1$ switching, reported in Fig. 4 (d), in which at least two antivortices were involved: the first annihilated the trapped vortex, and the subsequent one got trapped. The observed stratification could be the stroboscopic effect in the flux-flow state, determined by the ratio of the pulse length to the vortex time of flight. If so, this will not play a role when the vortex is manipulated by short pulses, which is the ultimate goal for such devices.

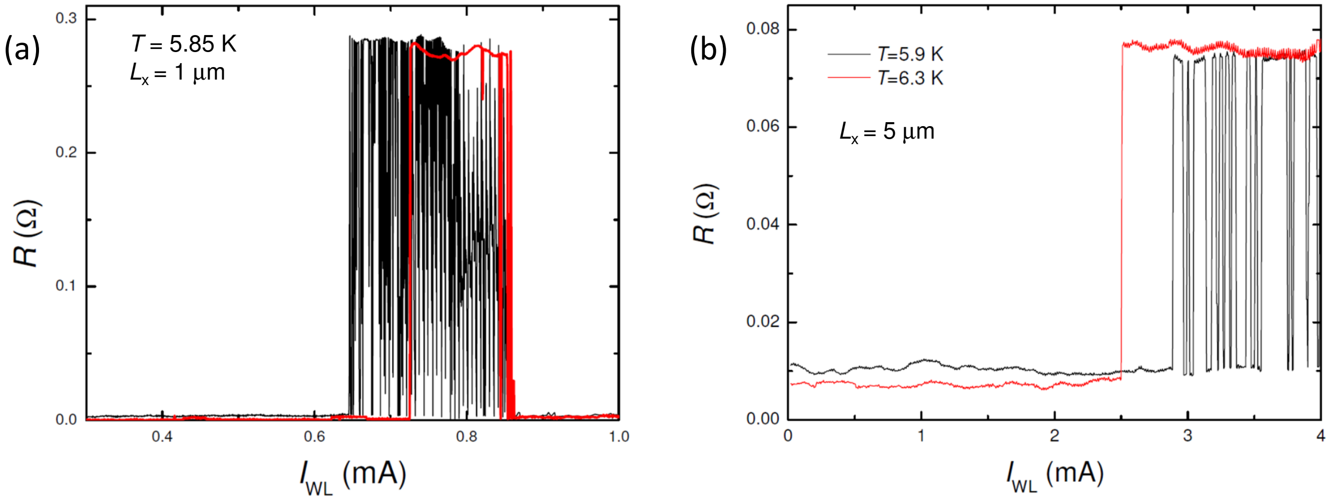

Supplementary Figure 7. **Demonstration of removal of switching current stratification.**
(a) Switching diagrams for the 1×1 μm^2 cell at a fixed $T = 5.85$ K, but measured with pulses of different duration, 0.33 ms (black) and 1 ms (red). (b) Switching diagrams for a $L_x = 5$ μm cell at the same pulse duration but different temperatures, $T = 5.9$ K (black) and 6.3 K (red). It is seen that the stratification is removed by changing the pulse duration, or temperature. The latter affects vortex time of flight by changing the viscosity.

VIII. Strategies for building AVRAM arrays

There are many potential hazards on the way from a single cell to a multi-cell RAM. The problems are caused by mutual crosstalk between cells, either via current sneak paths in a RAM array or via inductive coupling of cells and electrodes. To avoid this, some sort of isolation between WL and BL is needed. We have two possible strategies in mind. The first is based on a physical isolation with implementation of additional, electrically isolated control lines (CL's). Such a strategy is used in the existing RSFQ RAM. The implementation of CL's does not strongly affect the cell footprint but requires vertical stacking of layers. The alternative approach is based on logical (hardware) isolation. For example, in MRAM, the selection is achieved by adding a switching transistor for each cell. Unfortunately, there is no superconducting transistor with good enough isolation. However, recently we have demonstrated reconfigurable vortex-based superconducting diodes with high nonreciprocity [32]. Potentially, such programmable diodes can be employed to reduce sneak paths in a RAM bank. However, this comes at an expense of inevitable growth of complexity and footprint. At present, the second strategy looks more cumbersome. Nevertheless, diodes can be very instrumental for multiplexing.

IX. Crosstalk and AVRAM density

Apart from the size of a single cell, the AVRAM density can be also affected by the crosstalk between neighbor cells. The crosstalk can be caused either by vortex stray fields or by inductive coupling between electrodes.

We believe that the crosstalk via vortex stray fields should not be critical for ultimate miniaturization of AVRAM for two reasons. Firstly, stray fields are caused by the large demagnetization factor in planar geometry and their spatial extent is determined by the thickness of the film, which corresponds to the effective length of the magnetic dipole. It can be made very small, in the 10 nm range, greatly reducing stray fields at the estimated limit of miniaturization of about 100 nm. Secondly, in the mesoscopic case, when the size of the electrode becomes comparable or smaller than the Pearl length ($\lambda_P \simeq 500$ nm for our films [34]), the flux in the vortex is no longer quantized. The net flux decreases proportionally to the square of the ratio of the island size to the Pearl length, resulting in a corresponding reduction of stray fields. In the deep mesoscopic limit, $L_{x,z} \ll \lambda_P$, the vortex does not carry any flux at all, thus eliminating vortex stray fields.

There could also be an inductive crosstalk via control lines: the control-line current will induce some field at nearby cell rows as well. This type of crosstalk can be mitigated in two ways: 1) Improving the half-selection stability: if it is good enough the modest crosstalk field would not be critical. As demonstrated earlier [7], the half-selection stability of AVRAM cells can be very good. The observed cooperative WL+BL effect further improves the stability. 2) Proper geometrical design. Here the point is that planar structures respond only to the perpendicular component of magnetic field. The control line generates fairly uniform parallel fields above and below the CL, but highly nonuniform (almost singular) perpendicular field component at the edges, which decay at the scale of the film thickness. Therefore, the crosstalk via perpendicular field component can be made very short-range (~ 10 nm) with the corresponding minimal influence on the RAM density.
